# Importance of Resolving Fungal Nomenclature: the Case of Multiple Pathogenic Species in the *Cryptococcus* Genus

**DOI:** 10.1128/mSphere.00238-17

**Published:** 2017-08-30

**Authors:** Ferry Hagen, H. Thorsten Lumbsch, Valentina Arsic Arsenijevic, Hamid Badali, Sebastien Bertout, R. Blake Billmyre, M. Rosa Bragulat, F. Javier Cabañes, Mauricio Carbia, Arunaloke Chakrabarti, Sudha Chaturvedi, Vishnu Chaturvedi, Min Chen, Anuradha Chowdhary, Maria-Francisca Colom, Oliver A. Cornely, Pedro W. Crous, Maria S. Cuétara, Mara R. Diaz, Ana Espinel-Ingroff, Hamed Fakhim, Rama Falk, Wenjie Fang, Patricia F. Herkert, Consuelo Ferrer Rodríguez, James A. Fraser, Josepa Gené, Josep Guarro, Alexander Idnurm, María-Teresa Illnait-Zaragozi, Ziauddin Khan, Kantarawee Khayhan, Anna Kolecka, Cletus P. Kurtzman, Katrien Lagrou, Wanqing Liao, Carlos Linares, Jacques F. Meis, Kirsten Nielsen, Tinashe K. Nyazika, Weihua Pan, Marina Pekmezovic, Itzhack Polacheck, Brunella Posteraro, Flavio de Queiroz Telles, Orazio Romeo, Manuel Sánchez, Ana Sampaio, Maurizio Sanguinetti, Pojana Sriburee, Takashi Sugita, Saad J. Taj-Aldeen, Masako Takashima, John W. Taylor, Bart Theelen, Rok Tomazin, Paul E. Verweij, Retno Wahyuningsih, Ping Wang, Teun Boekhout

**Affiliations:** a Department of Medical Microbiology and Infectious Diseases, Canisius-Wilhelmina Hospital, Nijmegen, The Netherlands; b Centre of Expertise in Mycology Radboudumc/CWZ, Nijmegen, The Netherlands; c Science & Education, The Field Museum, Chicago, Illinois, USA; d Institute of Microbiology and Immunology, Faculty of Medicine, University of Belgrade, Belgrade, Serbia; e Department of Medical Mycology and Parasitology/Invasive Fungi Research Center (IFRC), Mazandaran University of Medical Sciences, Sari, Iran; f Unité Mixte Internationale Recherches Translationnelles sur l’Infection à VIH et les Maladies Infectieuses, Laboratoire de Parasitologie et Mycologie Médicale, UFR Pharmacie, Université Montpellier, Montpellier, France; g Department of Molecular Genetics and Microbiology, Duke University Medical Center, Durham, North Carolina, USA; h Veterinary Mycology Group, Department of Animal Health and Anatomy, Universitat Autònoma de Barcelona, Bellaterra, Barcelona, Spain; i Departamento de Parasitología y Micología, Instituto de Higiene, Facultad de Medicina, Universidad de la República, Montevideo, Uruguay; j Department of Medical Microbiology, Postgraduate Institute of Medical Education and Research, Chandigarh, India; k Mycology Laboratory, Wadsworth Center, New York State Department of Health, Albany, New York, USA; l Shanghai Key Laboratory of Molecular Medical Mycology, Shanghai Institute of Medical Mycology, Second Military Medical University, Shanghai, China; m Department of Dermatology, Changzheng Hospital, Second Military Medical University, Shanghai, China; n Department of Medical Mycology, Vallabhbhai Patel Chest Institute, University of Delhi, Delhi, India; o Medical School, Universidad Miguel Hernández, Alicante, Spain; p CECAD Cluster of Excellence, University of Cologne, Cologne, Germany; q Department I for Internal Medicine, University Hospital of Cologne, Cologne, Germany; r Center for Clinical Trials, University Hospital Cologne, Cologne, Germany; s Phytopathology Research, Westerdijk Fungal Biodiversity Institute, Utrecht, The Netherlands; t Department of Entomology and Plant Pathology, Faculty of Agriculture, Chiang Mai University, Chiang Mai, Thailand; u Department of Microbiology and Plant Pathology, Forestry and Agricultural Biotechnology Institute (FABI), University of Pretoria, Pretoria, South Africa; v Department of Microbiology, Hospital Severo Ochoa, Madrid, Spain; w University of Miami, NSF NIEHS Oceans and Human Health Center, Miami, Florida, USA; x Rosentiel School of Marine and Atmospheric Science, Division of Marine Biology and Fisheries, University of Miami, Miami, Florida, USA; y VCU Medical Center, Richmond, Virginia, USA; z Department of Medical Parasitology and Mycology/Cellular and Molecular Research Center, Urmia University of Medical Sciences, Urmia, Iran; aa Department of Clinical Microbiology and Infectious Diseases, Hadassah-Hebrew University Medical Center, Ein Kerem, Jerusalem, Israel; bb Department of Fisheries and Aquaculture, Ministry of Agriculture and Rural Development, Nir-David, Israel; cc Postgraduate Program in Microbiology, Parasitology and Pathology, Biological Sciences, Department of Basic Pathology, Federal University of Parana, Curitiba, Brazil; dd Australian Infectious Diseases Research Centre, School of Chemistry & Molecular Biosciences, University of Queensland, Brisbane, Australia; ee Unitat de Micologia, Facultat de Medicina i Ciències de la Salut, IISPV, Universitat Rovira i Virgili, Reus, Spain; ff School of BioSciences, BioSciences 2, University of Melbourne, Melbourne, Australia; gg Department of Bacteriology and Mycology, Tropical Medicine Institute Pedro Kouri, Havana, Cuba; hh Department of Microbiology, Faculty of Medicine, Kuwait University, Safat, Kuwait; ii Department of Microbiology and Parasitology, Faculty of Medical Sciences, University of Phayao, Phayao, Thailand; jj Mycotoxin Prevention and Applied Microbiology Research Unit, National Center for Agricultural Utilization Research, USDA-ARS, Peoria, Illinois, USA; kk Department of Laboratory Medicine, University Hospitals Leuven, Leuven, Belgium; ll Department of Microbiology and Immunology, KU Leuven - University of Leuven, Leuven, Belgium; mm Department of Microbiology and Immunology, University of Minnesota, Minneapolis, Minnesota, USA; nn Department of Medical Microbiology, College of Health Sciences, University of Zimbabwe, Harare, Zimbabwe; oo Malawi-Liverpool-Wellcome Trust, College of Medicine, University of Malawi, Blantyre, Malawi; pp School of Tropical Medicine, Liverpool, United Kingdom; qq Faculty of Medicine. University of Belgrade, Belgrade, Serbia; rr Institute of Public Health (Section of Hygiene), Università Cattolica del Sacro Cuore, Fondazione Policlinico Universitario Agostino Gemelli, Rome, Italy; ss Department of Communitarian Health, Hospital de Clínicas, Federal University of Parana, Curitiba, Brazil; tt Department of Chemical, Biological, Pharmaceutical and Environmental Sciences, University of Messina, Messina, Italy; uu IRCCS Centro Neurolesi Bonino-Pulejo, Messina, Italy; vv Centro de Investigação e de Tecnologias Agro-ambientais e Biológicas (CITAB), Universidade de Trás-os-Montes e Alto Douro (UTAD), Quinta dos Prados, Vila Real, Portugal; ww Institute of Microbiology, Università Cattolica del Sacro Cuore, Fondazione Policlinico Universitario Agostino Gemelli, Rome, Italy; xx Department of Microbiology, Faculty of Medicine, Chiang Mai University, Chiang Mai, Thailand; yy Department of Microbiology, Meiji Pharmaceutical University, Noshio, Kiyose, Tokyo, Japan; zz Mycology Unit, Microbiology Division, Department of Laboratory Medicine and Pathology, Hamad Medical Corporation, Doha, Qatar; aaa Japan Collection of Microorganisms, RIKEN BioResource Center, Koyadai, Tsukuba, Ibaraki, Japan; bbb Department of Plant and Microbial Biology, University of California Berkeley, Berkeley, California, USA; ccc Institute of Microbiology and Immunology, Faculty of Medicine, University of Ljubljana, Ljubljana, Slovenia; ddd Department of Medical Microbiology, Radboud University Medical Centre, Nijmegen, The Netherlands; eee Department of Parasitology, Faculty of Medicine, Universitas Indonesia, Jakarta, Indonesia; fff Department of Parasitology, School of Medicine, Universitas Kristen Indonesia, Jakarta, Indonesia; ggg Department of Microbiology, Immunology and Parasitology, Louisiana State University Health Sciences Center, New Orleans, Louisiana, USA; hhh Department of Pediatrics, Louisiana State University Health Sciences Center, New Orleans, Louisiana, USA; iii Institute of Biodiversity and Ecosystems Dynamics (IBED), University of Amsterdam, Amsterdam, The Netherlands; jjj Yeast Research, Westerdijk Fungal Biodiversity Institute, Utrecht, The Netherlands; University of Texas Health Science Center

**Keywords:** *Cryptococcus*, cryptococcosis, diagnostics, species delimitation, taxonomy

## Abstract

Cryptococcosis is a major fungal disease caused by members of the *Cryptococcus gattii* and *Cryptococcus neoformans* species complexes. After more than 15 years of molecular genetic and phenotypic studies and much debate, a proposal for a taxonomic revision was made. The two varieties within *C. neoformans* were raised to species level, and the same was done for five genotypes within *C. gattii*. In a recent perspective (K. J. Kwon-Chung et al., mSphere 2:e00357-16, 2017, https://doi.org/10.1128/mSphere.00357-16), it was argued that this taxonomic proposal was premature and without consensus in the community. Although the authors of the perspective recognized the existence of genetic diversity, they preferred the use of the informal nomenclature “*C. neoformans* species complex” and “*C. gattii* species complex.” Here we highlight the advantage of recognizing these seven species, as ignoring these species will impede deciphering further biologically and clinically relevant differences between them, which may in turn delay future clinical advances.

## Abstract (precis)

Cryptococcosis is a major fungal disease caused by members of the *Cryptococcus gattii* and *Cryptococcus neoformans* species complexes. After more than 15 years of molecular genetic and phenotypic studies and much debate, a proposal for a taxonomic revision was made.

## PERSPECTIVE

This perspective concerns the revision of the genus *Cryptococcus* in 2015 to recognize seven new species in what had been considered to be two species complexes of this important human-pathogenic fungus (1) and the more recent perspective (2) criticizing the 2015 revision. The following three main issues were raised (2). (i) The taxonomic proposal is premature. (ii) The new species cannot be identified using phenotypic tests alone. (iii) The new species names are confusing. The “2015 taxonomy paper” (1) has been highly cited, indicating that it fulfills a role in the scientific discussions on the taxonomy of the species complexes. At the recently held 10th International Conference on Cryptococcus and Cryptococcosis (ICCC10) (Foz do Iguaçu, Brazil, 26 to 30 March 2017), this matter was once more discussed, and ample evidence was provided that at least seven, and likely even more, species exist.

Cryptococcosis is an important fungal infection, globally affecting immunocompromised and immunocompetent humans and animals (3, 4). Annually more than 200,000 HIV-positive individuals develop cryptococcal meningitis with approximately 180,000 casualties (5). The phenotypic heterogeneity within the *Cryptococcus neoformans* species complex has been known for many years, beginning with the identification of four serotypes, serotypes A to D (6, 7). The discovery of an atypical clinical cryptococcal isolate led to the designation of a new variety named *C. neoformans* var. *gattii* (serotypes B and C) next to *C. neoformans* var. *neoformans* (serotypes A and D) (8, 9). The observation of the sexual cycle led to the description of *Filobasidiella neoformans* and *Filobasidiella bacillispora* (10–12). A third variety, *C. neoformans* var. *grubii*, was introduced in 1999 for serotype A strains, thus the variety *neoformans* became restricted to serotype D strains (13). In 2002, *C. neoformans* var. *gattii* was raised to species level, and the name* C. gattii* was given nomenclatural priority over the older name *C. bacillisporus* (14). At this stage, two species, *C. gattii* and *C. neoformans*, were recognized with the latter comprising two varieties, *neoformans* and *grubii*. The presence of diploid and aneuploid serotype A and serotype D hybrids (*C. neoformans* × *C. deneoformans*) has been known for a long time (7, 15–18), and they constitute 19 to 36% of the cryptococcal agents in southern Europe (19, 20). It is noteworthy that from a nomenclatural point of view, the type strain of *C. neoformans* CBS132 is a serotype AD hybrid (1, 17).

Morphology is a poor predictor to infer phylogenetic relationships of fungal isolates and particularly so for yeasts (21–27). Recently, the earlier name used to refer to the yeast morphology of *Cryptococcus* isolates was given priority over the teleomorphic name *Filobasidiella* (21, 22). The genus *Cryptococcus* in its current concept contains the dimorphic yeasts *C. amylolentus*, *C. bacillisporus*, *C. decagattii*, *C. deneoformans*, *C. deuterogatttii*, *C. neoformans*, *C. gattii*, and *C. tetragattii* (21, 22) and the filamentous species *C. depauperatus* and *C. luteus* (8, 22, 28, 29).

Molecular data revealed that the *C. neoformans* and *C. gattii* species complexes were unexpectedly genetically diverse (30). On the basis of four genes, it was calculated that *C. neoformans*/*C. deneoformans* separated from the *C. gattii* species complex 37 million years ago, *C. neoformans* and *C. deneoformans* separated 18.5 million years ago, and *C. gattii* and *C. bacillisporus* separated 9.5 million years ago (31). These divergence times might be older, as recent calculations based on genomic data fine-tuned the divergence time of the *C. neoformans*/*C. deneoformans* and the *C. gattii* species complex to 80 to 100 million years ago (32). The genomes of *C. deneoformans* and *C. neoformans* differ at ~10% of nucleotide positions (33). This difference is so large that the same phylogenetic groups have been found no matter which particular isolates were used and despite the increasing resolution of molecular typing tools, such as PCR-fingerprinting, amplified fragment length polymorphism (AFLP) fingerprinting, multilocus sequence typing (MLST), and whole-genome sequencing (WGS) (15, 30, 34–42).

Phenotypic, ecological, and geographical variation also supports creating species-level taxa in the *C. gattii* and *C. neoformans* species complexes (Table 1) (1, 43–67). For example, a recent study on virulence attributes such as capsule and melanin of members of the *C. gattii* species complex concluded with “These findings argue for increased acceptance of the new species and may be useful for informing diagnosis and prognosis in clinical infection” (50).

**TABLE 1:** Characteristics of pathogenic *Cryptococcus* species^a^

Characteristic	*C. neoformans*	*C*. *deneoformans*	*C*. *gattii*	*C*. *bacillisporus*	*C*. *deuterogattii*	*C*. *tetragattii*	*C*. *decagattii*
Genotype	AFLP1/VNI, AFLP1A/VNB/VNII, and AFLP1B/VNII	AFLP2/VNIV	AFLP4/VGI	AFLP5/VGIII	AFLP6/VGII	AFLP7/VGIV	AFLP10
Geographical distribution^b^	Worldwide (↑AFR)	Global (↑EUR)	Worldwide (↑ Asia, AUS, EUR)	Global (↑ California)	Worldwide (↑ AUS, NAM, SAM)	Sub-Saharan Africa and India	Latin America
Ecological preference	Bird droppings, soil, trees (1, 51–55)	Bird droppings, soil, trees (1, 51–55)	Trees (1)	Trees	Trees	?	?
Colonization	↑ in *Arabidopsis* *thaliana* compared to *C. deneoformans* (54)	↓ in *Arabidopsis* * thaliana* compared to *C. neoformans* (54)	ND	ND	ND	ND	ND
Animal infection	↑ Birds	?	↑ Mammals	Mammals	↑ Mammals	?	?
Susceptibility to antifungal drugs^c^	↑ GM MICs for AMB than *C. deneoformans* and interspecies hybrids (19, 48); ↑ GM MICs for 5FC compared to *C. tetragattii* (152)	↑ GM MICs for 5FC than *C. neoformans* and interspecies hybrids (48)	↑ GM MICs for FLZ, ITZ, and VCZ than *C. neoformans* (49)	No specific determinants	↑ GM MICs for 5FC, FLZ, VCZ, ITZ, PSZ, and ISA than *C. gattii* (44–46)	↓ GM MICs for 5FC compared to *C. neoformans* (152)	?
Clinical/host immune status	Mainly immunocompromised (↑HIV), but subgenotype VNIγ from immunocompetent subjects (84). ↑ meningitis	Immunocompromised and immunocompetent, ↑ cutaneous and elderly (153)	↑ Apparently healthy subjects, ↑ cryptococcoma	↑ HIV-positive subjects	↑ Apparently healthy subjects, ↑ pulmonary infections	↑ HIV-positive subjects	HIV-positive subjects
Capsule properties	↓ compared to *C. gattii sensu lato* (154)	ND	↑ compared to *C. neoformans* (154); ↑ compared to *C. bacillisporus*, *C. deuterogattii*, and *C. tetragattii* (50)	↑ compared to *C. neoformans* and *C. deuterogattii* (154)	↑ compared to *C. neoformans* (154); ↓ compared to *C. bacillisporus*, *C. gattii*, and *C. tetragattii* (48)	↑ compared to *C. neoformans* (154)	ND
Cell volume	ND	ND	↓ compared to *C. bacillisporus*, *C. deuterogattii*, and *C. tetragattii*; absence of giant cells (50)	ND	↑ compared to *C. bacillisporus*, *C. gattii*, and *C. tetragattii*;i ↑ giant cells (50)	↑ Giant cells (50)	ND
Thermotolerance	↑ Growth rate at 37°C (154)	↓ Growth rate at 37°C (154)	↓ Growth rate at 37°C (154); intermediate compared to *C. bacillisporus*, *C. deuterogattii*, and *C. tetragattii* (50)	↓ Growth rate at 37°C (154); ↓ compared to *C. gattii*, *C. deuterogattii*, and *C. tetragattii* (50)	↓ Growth rate at 37°C compared to *C. neoformans* (154); ↑ compared to *C. gattii*, *C. bacillisporus*, and *C. tetragattii* (50)	↓ compared to *C. gattii*, *C. bacillisporus*, and *C. deuterogattii* (50)	ND
Melanin	↑ compared to *C. gattii sensu lato* (154)	ND	↓ compared to *C. neoformans* (154)	↓ compared to *C. neoformans* (154)	↓ compared to *C. neoformans* (154)	↓ compared to *C. neoformans* (154)	ND
Virulence in *Drosophila* * melanogaster* model	ND	ND	↓ compared to *C. bacillisporus* (154)	↑ compared to *C. gattii*, *C. deuterogattii*, and *C. tetragattii* (154)	↓ compared to *C. bacillisporus* (154)	↓ compared to *C. bacillisporus* (154)	ND
RNAi pathway^d^	Present (65)	Present (65)	Present (65)	Present (65)	Lost (65)	Present (65)	ND
Mycophenolic acid	Sensitive (66)	Sensitive (66)	Sensitive (66)	Sensitive (66)	Sensitive (66)	Not sensitive (66)	ND
Growth on the following medium:							
CGB	Yellowish	Yellowish	Blue	Blue	Blue	Blue	Blue
CDBT	Pale colonies with no apparent color effect on the medium (155)	Colonies bright red, medium bright orange (155)	ND	ND	ND	ND	ND

a Overview of characteristics of the pathogenic *Cryptococcus* species, using data from Hagen et al. (1) and updated where indicated with reference numbers. See reference 1, including its supplemental data, for more-detailed phenotypic information. A question mark indicates that the specific item is unknown. ↑, higher or increase in; ↓, lower or decrease in; ND, not determined.

b Abbreviations: AFR, Africa; EUR, Europe; AUS, Australia: NAM, North America; SAM, South America.

c Abbreviations: GM, geometric mean; AMB, amphotericin B; 5FC, 5-fluorocytosine; FLZ, fluoconazole; ISA, isavuconazole; ITZ, itraconazole; PSZ, posaconazole; VCZ, voriconazole.

d RNAi, RNA interference.

Genetic methods revealed that intraspecies crosses between *C. neoformans* and *C. deneoformans* isolates showed a higher spore viability compared to *C. deneoformans* × *C. neoformans* interspecies crosses (33). Twenty-three quantitative trait loci were identified from the analysis of interspecific crosses involved in virulence-associated and azole-resistant phenotype differences between both species (61), and the observed postzygotic isolation mechanisms were explained by Bateson-Dobzhansky-Muller incompatibility affecting basidiospore viability in interspecific crosses (62). Mitotic recombination, causing chromosomal loss and crossing over, seems a further genetic separation mechanism between both species (63). One study indicated that *C. neoformans* (cited as serotype A strains) reproduced mainly clonally, whereas *C. deneoformans* (cited as serotype D strains) showed recombination. Moreover, genomic differences and MLST analysis separated both species (64).

Cryptococcosis is usually diagnosed by microscopy, histopathology, culture, and serology, including lateral flow assays, and by molecular assays (Table 1) (68–92), all of which allow straightforward identification of unknown environmental and clinical cryptococcal isolates. Importantly, the matrix-assisted laser desorption ionization–time of flight mass spectrometry (MALDI-TOF MS) approach can reliably identify the recognized species of *Cryptococcus* (that may have been cited as genotypes) (1, 93, 94). Kwon-Chung and coworkers (2) questioned the usefulness of MALDI-TOF MS for the separation of the new species and the hybrids, suggesting that only score values of ≥2.0 indicate a reliable species identification. However, several studies show that yeast and even filamentous fungal isolates can be reliably identified with a score value of ≥1.7 (95–97), and this is acknowledged in the current Bruker guidelines. The identification of *Cryptococcus* isolates by MALDI-TOF MS yields comparable results or even outperforms the identification methods used for *Candida*, *Geotrichum*, *Malassezia*, and *Trichosporon* isolates.

Kwon-Chung and coworkers (2) questioned the phylogenetic methods that were used to delimit the seven species. Yeast biodiversity research has changed from a discipline driven mainly by phenotype to a discipline based largely on molecular variation (98, 99). Molecular phylogenetic analyses of many species complexes of fungi have resulted in the recognition of new species based on molecular variation. An early example was the recognition and description of the human-pathogenic genus *Coccidioides* based solely on molecular variation (100). New, molecularly defined species are common in yeasts and include the recognition of many “cryptic,” “sibling,” and “sister” species. Examples are *Saccharomyces eubayanus*/*S. uvarum* (101), *Candida albicans*/*C. africana/C. stellatoidea* (102–106), *Candida auris*/*C. haemulonii/C. duobushaemulonii* (107–112), *Candida glabrata*/*C. nivariensis*/*C. bracarensis* (103, 113–115), *Candida parapsilosis*/*C. orthopsilosis*/*C. metapsilosis* (103, 116), *Malassezia furfur* that now comprises 16 species (117–119), *Trichosporon cutaneum* with at least 10 species (120, 121), the *Aspergillus fumigatus* complex (122–124), *Coccidioides immitis*/*C. posadasii* (100), and *Paracoccidioides brasiliensis/P. lutzii* (125). Although this listing is far from complete, it underlines the impact of molecular taxonomic studies for clinically important yeasts and molds.

Kwon-Chung and coworkers (2) suggested that methods employed in the 2015 taxonomic proposal are not appropriate because they have been developed for sexually reproducing organisms. One of the first applications of molecular recognition of species was with a fungus that has yet to reveal its sexual morphology, *Coccidioides* (100). Furthermore, *Cryptococcus* has a sexual cycle and clearly can reproduce both sexually and asexually. Moreover, the methods used have been applied to identify species-level lineages in asexual taxa (126–134). Methods using branch length differences to identify thresholds between intra- and interspecific distances (such as the coalescence-based general mixed Yule coalescent method) potentially underestimate species diversity in asexual species, since sexual species are separated by larger genetic gaps than asexual species (135). Individual methods for species delimitation based on molecular data have been shown to either oversplit or underestimate species diversity under specific circumstances (136); understanding the performance of each method is still in its infancy given the recent and rapid development of this field of research. Therefore, three independent approaches were used to delimit species boundaries within the *C. neoformans*/*C. gattii* species complexes. In addition, DNA-based approaches were congruent with, for example, MALDI-TOF MS-based data. Sampling of additional loci would certainly be useful, as well as the addition of further genomic data sets. However, studies of other microorganisms repeatedly show that additional loci will either confirm clades found or reveal the presence of new ones. Thus, species delimitation for the seven etiologic agents of cryptococcosis was minimal and conservative (1). Most, if not all, studies that used whole-genome data published before the 2015 taxonomy paper (cited in reference 1), and thereafter, e.g., Farrer and coworkers (36) and those presented at ICCC10 (42, 43, 137–139) identified the same species clades.

The insights that resulted in the 2015 taxonomy proposal (1) were elaborated, presented, and discussed at several related meetings from ICCC4 (London, United Kingdom, 1999) to ICCC10 (Foz do Iguaçu, Brazil, 2017). At ICCC6 (Boston, MA, USA, 2005), a debate entitled “*Cryptococcus neoformans*: one, two or more species” was held. Two different opinions were presented, namely, for two species or multiple species (at that time, six species). The community strongly supported the name *C. neoformans* for serotype A strains that are clinically important. The type strain of *C. nasalis* belongs to serotype D (15); hence, it had nomenclatural priority. However, the community leaders present at ICCC6 to ICCC8 were strongly against the use of this name. Therefore, *C. deneoformans* was proposed for this clade at ICCC6, as it shows affinity with the epithet *neoformans* and serotype D (*de-neoformans*). The name *C. gattii* received renewed attention, as it was reported as the cause of a number of major outbreaks (35, 140, 141). The rules of fungal nomenclature do not allow this name to be used for a clade other than the one containing the type strain (and ex-type strain). The clade referred to as AFLP4/VGI represents *C. gattii*, and the AFLP5/VGIII clade is *C. bacillisporus*. Three other consistently observed clades in the *C. gattii* species complex were named using “*gattii*” in part of the epithet in order to keep reference to the name “*gattii*.”

The taxonomy of the species complexes is complicated by various interspecies hybrids (16, 20, 142–147). Hybrids occur among many yeast genera, such as *Saccharomyces*, where well-recognized species form hybrids and even triple hybrids (147–150). For *Saccharomyces* hybrids, a conventional nomenclature has been proposed (150). The species that contribute to the hybrid will be given in alphabetic order, and in cases where the genomic contribution is known, this will be indicated. For instance, the type strain of *S. bayanus* CBS380 is written as *S. cerevisiae* <1% × *S. eubayanus* 37% × *S. uvarum* 63%. This convention is also applicable to the genus *Cryptococcus*. The hybrid type strain of *C. neoformans* can be thus described as *C. deneoformans* × *C. neoformans*.

## FOLLOWING THE RULES OF THE INTERNATIONAL CODE OF NOMENCLATURE

The naming of fungi is governed by the *International Code of Nomenclature for Algae*, *Fungi*, *and Plants*, and naming fungi is based on a number of principles (151). Among them, the priority principle implies that the oldest validly given name should be applied to an organism and that the phylogenetic position of the type that determines the name has to be given to a certain clade at a specific taxonomic level. Thus, when a validly described species name exists for a certain species, that name must be used. This was the case for the species that were reinstalled as *C. gattii*, *C. bacillisporus*, and in fact also for *C. deneoformans* (see above).

## SUMMARY

The main advantage of recognizing seven species rather than just two “species complexes” (*viz*., *C. gattii sensu lato* and *C. neoformans sensu lato*) is that researchers and clinicians will be stimulated to search for further phenotypic and genetic differences and similarities between the recognized species. This stimulation of research has already yielded new genetic, molecular, and phenotypic features, including differences in drug susceptibility (Table 1). The recognized species can be identified using a diverse array of molecular diagnostics and MALDI-TOF MS, and some of them can already be identified by phenotypic means. Ignoring the species impedes deciphering the differences among them, which may delay future clinical advances. Finally, it is apparent that more species seem to occur within *Cryptococcus*, e.g., the Botswana lineage within *C. neoformans* (18, 137–139).
